# **Sugar-Sweetened Beverage Taxes Are a Sweet Deal: Improve Health**, **Save Money**, **Reduce Disparities**, **and Raise Revenue**

**DOI:** 10.1093/jncics/pkaa075

**Published:** 2020-08-25

**Authors:** Zachary J Ward, Steven L Gortmaker,

**Affiliations:** 1 Center for Health Decision Science, Harvard T.H. Chan School of Public Health, Boston, MA, USA; 2 Department of Social and Behavioral Sciences, Harvard T.H. Chan School of Public Health, Boston, MA, USA

The obesity epidemic continues to grow in the United States and is projected to affect nearly half of adults by 2030, with large disparities by income and race and ethnicity (1). These disparities are being starkly exposed by the current SARS-CoV-2 pandemic, with increasing evidence that obesity is a risk factor for increased morbidity and mortality from coronavirus disease 2019 (COVID-19) (2,3). However, although it typically receives less attention, the long-running epidemic of obesity-related chronic disease in the United States has also claimed many lives. In addition to cardiovascular diseases and diabetes, obesity is a risk factor for multiple cancers, which comprise a substantial burden of potentially avoidable morbidity and mortality in the US population. Effective policies and programs to prevent obesity are thus critical to improve population health, because obesity is a risk factor for both chronic and infectious disease outcomes.

In this issue of the Journal, Du et al. (4) present a cost-effectiveness analysis (CEA) of a $0.01 per ounce sugar-sweetened beverage (SSB) excise tax on obesity-related cancer outcomes, finding that it would be a cost-effective policy to prevent obesity-related cancers and reduce disparities in cancer incidence. These findings are broadly similar to other SSB tax CEAs that find that a national SSB tax would be highly cost-effective and likely even cost-saving—that is, improving health outcomes while also saving more in healthcare spending than it would cost to implement—when broader health outcomes are taken into account (5–7). The current analysis by Du et al. (4) focuses only on cancer outcomes, which do not capture the full health benefits of an SSB tax. However, this more focused analysis of obesity-related cancers should be of interest to the wider cancer community —a group of stakeholders that is increasingly involved in obesity prevention efforts. Given the increased risk of cancer incidence associated with excess weight and disparities in cancer incidence, obesity prevention plays an important role in cancer control efforts and planning.

In addition to only focusing on cancer outcomes, the modeling analysis by Du et al. (4) makes other assumptions that may lead to conservative estimates of the cost-effectiveness of an SSB tax. For example, as a closed cohort model, the impact on future adults (ie, current children) is not taken into account. Because the effect of an SSB tax would be expected to persist as long as the policy is in place, one would expect benefits to accrue from preventing excess weight gain for future adults in the United States as well.

The authors also assume a very low own-price elasticity of demand for SSBs. An authoritative review by Powell et al. (8), focusing on better-designed studies (generally using demand system estimation), found an average elasticity of -1.21, and recent estimates from evaluating the impact of the SSB tax in Philadelphia, Pennsylvania, find an even larger effect of -1.7 (9). The -0.66 used in this paper [Du et al. (4)] thus likely underestimates the impact of an SSB excise tax. In that sense, the sensitivity analysis presented in Du et al. (4) in supplementary table 8 (available online) may be a more realistic estimate. Here, the authors assume an own-price elasticity of -1.14 and find with this assumption that “the estimated health gains doubled, and the policy became cost-saving”.

Nevertheless, although the specific modeling assumptions of any analysis can (and should) be interrogated, the overarching findings from various models (all with different assumptions and structures) point to the potential for SSB taxes to be a highly cost-effective policy that meaningfully improves population health.

In general, one can consider the impact of a policy in terms of its effect (what is the individual-level effect size), cost (what are the net costs of implementing the policy), and reach (how many people would be impacted). For example, a cost-effective policy may have a large effect and small cost, but if it only impacts a small number of individuals, it will not have a large impact on population health. In contrast, the broad reach of an SSB tax means that it scores highly on all 3 metrics, having a relatively large impact on the general population at low cost.

In addition to improving population health overall, the analysis by Du et al. (4) finds that an SSB tax would also reduce disparities in cancer incidence, with women and minorities estimated to have the largest health gains. SSB taxes appear to impact disparities in multiple ways. For example, racial and ethnic minorities and low-income populations tend to have higher consumption of SSBs (10) and may be more price sensitive than other populations (11,12). As cancer incidence and mortality are generally higher among lower socioeconomic and non-Hispanic Black individuals, in part because of differences in risk factor exposures (13), such policies are needed that can reduce disparities while improving population health.

Lastly, in addition to improved health and reduced healthcare spending, an SSB tax would raise revenue. Whereas tax revenue is not considered a benefit in economic evaluation from the societal perspective, from the government perspective, tax revenue is certainly an important consideration, especially given the looming fiscal crises many states face as a result of the current COVID-19 pandemic. Furthermore, earmarking revenue for specific activities has been found to increase public support for SSB taxes (14). Indeed, the recently implemented SSB tax in Philadelphia, Pennsylvania, was introduced with the explicit goal of financing universal prekindergarten and deliberately not framed as a health intervention (15). With the recent severe cuts to many education budgets, an SSB tax could similarly provide much-needed financing for other areas around the country.

SSB taxes are thus an attractive policy: in addition to the useful revenue that they raise (especially important during the current fiscal crisis), they improve population health, reduce future healthcare costs, and reduce disparities. Why then are they not more widespread? Assertive lobbying from the beverage industry is a large obstacle (16). A common argument employed against SSB taxes is that they are regressive, in that people with less money would pay a larger proportion of their income to the tax. However, with a price elasticity likely greater than -1.0 (8,9), empirical evidence suggests that people will be spending less money overall on a product that harms them. Moreover, this view of the regressive nature of SSB taxes is too narrow. The future savings in health-care costs as a result of the tax would dwarf any increased spending now. Indeed, the analysis by Du et al. (4) finds that the SSB tax, evaluated only for its impact on cancer, would be cost-saving for low-income individuals, and cost-saving overall with an elasticity assumption of -1.14. Thus, with disproportionate benefits likely accruing to low-income populations, the taxes are not in fact regressive but are actually progressive policy.

As states and local governments consider ways to navigate the current health and fiscal crisis brought on by the COVID-19 pandemic, an SSB tax offers the potential to provide much-needed funding while also improving the long-term health of the population and reducing health-care costs and disparities.

## Notes

**Author contributions**: ZW: Drafted initial manuscript. All authors: Drafting, revising, editing manuscript.

**Disclosures**: The authors have no conflicts of interest to declare.

## Data Availability Statement

Not applicable.
